# Telemedicine in pediatrics: a critical narrative review of innovations, limitations, and future priorities

**DOI:** 10.1007/s00431-026-07262-1

**Published:** 2026-08-01

**Authors:** Gianvincenzo Zuccotti, Ilaria Anna Maria Scavone, Erika Cordaro, Virginia Rossi, Valeria Calcaterra

**Affiliations:** 1 Department of Biomedical and Clinical Science, University of Milano, c/o, Buzzi Children’s Hospital, Via Castelvetro N. 32, Milan, 20254 Italy; 2 Department of Pediatrics, Buzzi Children’s Hospital, Milan, Italy; 3 Pediatric and Adolescent Unit, Department of Internal Medicine and Therapeutics, University of Pavia, P.le Golgi 2, Pavia, 27100 Italy

**Keywords:** Pediatric telemedicine, Telehealth, Digital health, Remote monitoring, Pediatric chronic diseases, Virtual care

## Abstract

This narrative review synthesizes recent technological and organizational developments in pediatric telemedicine, maps their principal clinical applications, and critically discusses implementation, safety, equity, and future priorities. This narrative review used a transparent, non-systematic literature-search approach in PubMed/MEDLINE, Scopus, and Google Scholar, considering publications available through April 2026. Searches were intended to identify and contextualize relevant literature across pediatric settings rather than to provide an exhaustive, reproducible systematic evidence synthesis. No formal risk-of-bias assessment, meta-analysis, or certainty-of-evidence grading was undertaken; therefore, findings are interpreted cautiously and according to the maturity and consistency of the available evidence. Evidence suggests that telemedicine can support follow-up, access to specialist care, and family engagement in several pediatric chronic-care pathways, particularly diabetes and asthma. However, the evidence base is heterogeneous across specialties, frequently relies on observational or implementation studies, and is less mature for acute assessment, neonatal and rehabilitation uses, wearables, and AI-enabled tools.

*Conclusion*: Telemedicine should be considered a complementary component of pediatric healthcare rather than a replacement for in-person assessment. Hybrid models may support accessibility, continuity, and sustainability when embedded in structured, patient-centered, equitable, and clinically appropriate pathways.

**Table:** 

**What is Known:** • *Telemedicine expanded rapidly in pediatrics during the COVID-19 pandemic and is now used across many specialties.* • *It can improve access, continuity of care, remote monitoring, and family engagement, especially in chronic and complex conditions.*	
**What is New:** • *This review summarizes recent technological and organizational innovations, including wearables, mHealth, EHR integration, AI, and hybrid care models.* • *It highlights current limits and future requirements for safe, equitable, and sustainable integration into routine pediatric care.*

**What is Known:**

• *Telemedicine expanded rapidly in pediatrics during the COVID-19 pandemic and is now used across many specialties.*

• *It can improve access, continuity of care, remote monitoring, and family engagement, especially in chronic and complex conditions.*

**What is New:**

• *This review summarizes recent technological and organizational innovations, including wearables, mHealth, EHR integration, AI, and hybrid care models.*

• *It highlights current limits and future requirements for safe, equitable, and sustainable integration into routine pediatric care.*

## Introduction

Telemedicine refers to the remote delivery of clinical services, including synchronous or asynchronous teleconsultation, telemonitoring, teleassistance, and virtual visits [1]. Telehealth is a broader term encompassing telemedicine together with health education, administrative services, and other digitally enabled care processes [1–3]. Remote patient monitoring (RPM) captures patient- or family-generated health data outside the clinical setting; mHealth and eHealth refer to mobile- and digital-health tools; digital therapeutics are evidence-based software interventions intended to prevent, manage, or treat a condition; and AI-enabled care includes algorithm-supported prediction, triage, or decision support [1–4]. These categories overlap but differ in clinical purpose, regulatory status, and evidentiary requirements [1–4].

Its use expanded rapidly during the COVID-19 pandemic and has since evolved into an important component of pediatric care [2, 3, 5, 6].

Pediatric telemedicine differs from adult telemedicine because clinical assessment depends on age, developmental stage, communication abilities, and caregiver participation [5, 7]. Parents and caregivers frequently support symptom reporting, remote examination, device management, and therapeutic decision-making [1, 3]. Therefore, these approaches require careful attention to clinical appropriateness, privacy, informed consent, safety, and equity of access.

However, telemedicine cannot fully replace face-to-face pediatric care. Certain conditions still require direct physical examination, growth and developmental assessment, or diagnostic procedures that cannot be reliably performed remotely [8]. Consequently, telemedicine should be integrated into broader pediatric care pathways according to clinical needs and patient characteristics [9].

This narrative review aims to summarize recent technological and organizational innovations in pediatric telemedicine, describe its main clinical applications across different pediatric settings, and discuss future perspectives for its safe, effective, and equitable integration into routine pediatric care. Addressing a practical gap not fully covered by previous condition-specific or modality-specific reviews, it integrates clinical applications with key implementation requirements, including caregiver roles, safety, equity, interoperability, and the emerging use of artificial intelligence. Rather than providing a systematic estimate of effect, the review offers a critical cross-specialty overview of areas in which pediatric telemedicine is established, those in which the evidence remains preliminary, and the conditions required for its safe implementation in routine care.

## Methods

This narrative review provides a transparent, non-systematic overview of pediatric telemedicine, addressing technological innovations, clinical applications, benefits, limitations, and future priorities. We searched PubMed/MEDLINE, Scopus, and Google Scholar for studies and reviews available through April 2026 using the following core concept: (telemedicine OR telehealth OR “virtual care” OR telemonitoring OR “remote patient monitoring” OR mHealth OR eHealth OR “digital health” OR “artificial intelligence”) AND (pediatric* OR paediatric* OR child* OR adolescen* OR neonatal*). Searches were supplemented by reference-list screening and targeted retrieval of relevant clinical guidelines and consensus documents.

Eligible English-language sources included pediatric randomized controlled trials, observational studies, systematic reviews, meta-analyses, consensus documents, and clinical guidelines. We excluded adult-only studies, purely technical reports without pediatric clinical relevance, conference abstracts, and reports with insufficient methodological detail. The searches and supplementary retrieval procedures identified 1150 potentially relevant records. After removal of duplicates and clearly irrelevant publications, 500 titles and abstracts were screened, and 182 sources were assessed in greater detail. A total of 134 publications were retained to inform the narrative synthesis. Two authors independently screened titles/abstracts and full texts, resolving disagreements through discussion with a senior author. Findings were charted narratively by clinical setting, technology, implementation issue, and evidentiary maturity.

As this was a narrative rather than systematic review, we did not use a protocol-driven systematic-search framework, PRISMA flow diagram, quantitative synthesis, formal risk-of-bias assessment, or certainty-of-evidence grading. Given the non-systematic nature of the search, the reported numbers should be considered estimates. Therefore, study inclusion should not be interpreted as an endorsement of methodological quality, and heterogeneity in populations, interventions, comparators, terminology, and outcomes limits direct cross-study comparisons. Evidence-maturity descriptors are used only to orient readers to the consistency and clinical development of the literature; they do not represent formal evidence grades. Accordingly, findings are presented as evidence-informed rather than definitive.

## Recent novelties in pediatric telemedicine

Pediatric telemedicine has progressively evolved from isolated teleconsultation services into broader digital-health ecosystems integrating telemedicine platforms, wearable technologies, remote monitoring systems, mobile applications, and electronically connected care pathways [4, 10].

### Technological innovations

Clinical readiness varies across technologies. For this review, established tools are those integrated into defined care pathways with relatively consistent pediatric evidence (e.g., CGM-linked diabetes follow-up); emerging tools have promising but limited clinical evidence (e.g., selected home monitoring and digital therapeutics); consumer tools are not equivalent to clinical devices; and investigational AI systems require additional validation, governance, and regulatory scrutiny.

#### Dedicated telemedicine platforms

Telemedicine platforms are increasingly integrated into pediatric healthcare, supporting multidisciplinary care for children with chronic and complex conditions in geographically challenging settings. In pediatric diabetes, digital platforms such as CareLink, Clarity, Glooko, and t:connect improve remote glucose and insulin-pump monitoring during follow-up visits [11]. Home telecare programs also support pediatric home management through telemonitoring kits and continuous communication with healthcare teams, improving family satisfaction and preference for home hospitalization [12]. In pediatric palliative care, telemedicine platform facilitate communication, symptom assessment, follow-up, and multidisciplinary coordination for children with serious conditions [13]. However, remote evaluation remains limited when a detailed physical examination is required [12, 14]. Its implementation also depends on internet access, updated devices, digital literacy, and family familiarity with technology [11, 15].

#### Wearable and home-based monitoring devices

Wearable and home-based technologies are increasingly used in pediatric telemedicine to collect health data outside hospital settings, supporting monitoring of chronic diseases through sensors, connected devices, symptom trackers, and mobile systems [16]. These tools can monitor parameters such as heart rate, glucose, oxygen saturation, activity, sleep, symptoms, and treatment adherence, providing longitudinal clinical information. T1D represents one of the most advanced examples of wearable-enabled telemedicine. Insulin pumps, continuous glucose monitoring (CGM) systems, and cloud-based data-sharing platforms allow real-time review of glycemic patterns and more targeted treatment adjustments between traditional clinical encounters. Hybrid closed-loop systems can automate insulin delivery using CGM data and algorithms [17] while large CGM datasets support predictive analytics and hypoglycemia prediction [18]. Similar approaches have been developed for pediatric respiratory diseases using exercise trackers, sleep monitors, smart inhalers, and respiratory-monitoring systems [19]. WEARCON, a multidimensional wearable home-monitoring strategy integrating activity trackers, handheld spirometry, smart inhalers, and cardiorespiratory monitoring, identified uncontrolled asthma in 88.9% of children [20], and the BREATHE platform integrates smartwatch, spirometry, medication, geolocation, and pollution data for longitudinal asthma monitoring [19]. Clinical readiness varies: CGM and insulin-pump ecosystems are established clinical tools, whereas many consumer-grade wearables and app-linked devices require pediatric validation for accuracy, safety, interoperability, privacy, and appropriate clinical use.

However, device accuracy and reliability remain variable, especially with consumer-grade tools. Martinko et al. [21] reported considerable heterogeneity and limited precision in wearable devices for physical-activity monitoring in children.

#### Mobile health applications

mHealth applications are increasingly used in pediatrics for chronic disease management, self-monitoring, health education, treatment adherence, and caregiver support [10, 22]. Examples of widely available consumer platforms include Apple Health, Samsung Health, Google Fit, Garmin Connect, Yazio, MyFitnessPal, Calm, and Headspace; however, their availability or use in the general population should not be interpreted as evidence of pediatric clinical validity or effectiveness [23]. In pediatric obesity, interventions combine apps, wearable devices, SMS reminders, telecoaching, and self-monitoring systems [22, 24]. The SMARTFAMILY intervention integrated family-based goals, feedback, and behavioral strategies, although without significant improvement in physical activity or diet outcomes [25]. Telehealth interventions for obesity may still improve accessibility while achieving outcomes comparable to conventional care [26]. In pediatric asthma, digital interventions improved treatment adherence in 87% of studies, particularly when apps promoted active engagement [27]. Self-management applications with proactive clinical interaction were associated with reduced healthcare utilization [28]. In juvenile idiopathic arthritis, eHealth and mHealth tools supported symptom management and long-term care despite high heterogeneity across studies [10].

Digital tools are also increasingly used in pediatric mental health and neurodevelopmental disorders [29]. In autism spectrum disorder (ASD), telemedical tools allowed caregivers to apply behavioral interventions remotely under supervision [30]. EndeavorRx represents one of the first FDA-authorized prescription digital therapeutics for pediatric ADHD, delivering cognitive training through a game-based mobile platform [31]. However, evidence remains limited by small populations, short follow-up, heterogeneous outcomes, declining engagement, digital inequalities, privacy concerns, and inconsistent long-term efficacy [30, 32, 33].

#### Integration with electronic health records

Integration between telemedicine systems and EHRs supports remote data collection, longitudinal monitoring, multidisciplinary coordination, and incorporation of patient-generated data into pediatric workflows. Jung et al. [4] highlighted that integrated ICT systems allow access to longitudinal patient information within unified digital environments. Integration of PGHD is particularly relevant in chronic disease monitoring. Foster et al. [16] emphasized that RPM systems are clinically useful when collected data can be visualized, documented, and incorporated into EHR workflows, although workflow fragmentation remains a concern. T1D is among the most developed examples of EHR-integrated telemedicine because glucose-monitoring systems, insulin pumps, and cloud platforms support remote review of patient-generated data [11]. Within the T1DX-QI collaborative, centers used platforms such as CareLink, Dexcom Clarity, Glooko, and Tandem t:connect during telemedicine visits, although full interoperability remained limited [11].

Other examples include digital home-care systems combining telecommunication, wearable devices, caregiver portals, and remote documentation tools to support coordination among hospital teams, community providers, and families [12, 34]. Integration of Zoom within the Epic EHR platform to facilitate virtual visits through patient portals [35], is retained only as an illustrative implementation model, not as central pediatric effectiveness evidence.

Despite these advances, multiple studies continue to report fragmented data systems, lack of standardized workflows, and cybersecurity concerns as major barriers to full integration [11, 16, 33].

#### Artificial intelligence and clinical decision-support tools

Artificial intelligence (AI), machine learning (ML), and clinical decision-support systems are emerging components of pediatric digital health, with applications in predictive analytics, automated alerts, risk stratification, precision medicine, and clinical decision-making [4]. Their clinical value depends on data quality, external validation, explainability, regulatory oversight, cybersecurity, and clear allocation of medico-legal responsibility; they should support, rather than replace, clinical judgment [4]. Their implementation must also comply with evolving regulatory frameworks for AI-enabled medical software, including software as a medical device (SaMD) requirements, and should be supported by prospective clinical validation before widespread adoption.

Several applications have been described in pediatric telemedicine. Machine-learning models may predict childhood asthma persistence [36], while automated insulin-delivery systems continuously process CGM data to optimize glycemic control [17]. Dave et al. [18] developed a real-time hypoglycemia prediction model using CGM data from young patients with type 1 diabetes, achieving sensitivities above 91% and specificities over 90%. AI-enhanced tools are also emerging in developmental and psychosocial care through adaptive applications, serious games, digital coaching, and chatbot-supported interventions [37]. Overall, AI-based pediatric systems remain investigational in most settings because of heterogeneous datasets, limited external validation, potential algorithmic bias, uncertainty regarding explainability, and unresolved questions of clinical accountability.

### Organizational innovations

#### Hybrid models combining in-person and remote care

Hybrid pediatric care models combine remote and in-person care according to clinical complexity and family needs [26, 38]. They are particularly useful in obesity, diabetes, nutrition, and chronic disease management, where some components can be delivered remotely while growth assessment, diagnostic work-up, or direct examination require in-person evaluation. Tele-nutrition models may reserve in-person visits for these assessments while dietary counseling and follow-up are conducted remotely [39]. Similarly, obesity programs combine telehealth counseling and app-based self-monitoring with periodic in-person evaluations [24]. Hybrid pathways should specify eligibility criteria, red flags, escalation procedures, documentation requirements, and responsibility for follow-up.

#### Digital triage systems

Digital triage systems are increasingly used to determine the urgency of in-person evaluation, guide referrals, and optimize healthcare resources [38]. Telephone triage, videoconferencing, patient portals, and messaging systems may support decisions regarding home management, outpatient assessment, or urgent care [40]. Patel et al. [35] implemented an Epic-integrated color-coded workflow to classify patients for telemedicine, in-person evaluation, or deferred care. Safe implementation requires written triage criteria, clear red flags requiring in-person assessment, emergency instructions, defined escalation pathways, and documentation of clinical responsibility. Limitations include incomplete remote examination, variable caregiver participation, and technological barriers [38, 41].

#### Remote follow-up pathways

Remote follow-up pathways are widely used in postoperative care, palliative care, chronic disease management, diabetes, obesity, and home-based care programs [13, 42]. The NSIPP model combined home insulin-pump use with specialist assessment and structured diabetes education [43]. In asthma care, the Virtual Asthma Clinic replaced 50% of routine visits while maintaining asthma control [44]. mHealth postoperative systems supported wound assessment, symptom monitoring, and family communication after discharge [42]. Patel et al. [35] reported that most telemedicine encounters during implementation were follow-up or postoperative visits. Remote follow-up may improve continuity of care and reduce unnecessary hospital access when patients are clinically eligible and there is a reliable route back to in-person assessment [13]. Similar approaches are used in obesity and tele-nutrition programs through scheduled video consultations and remote dietary counseling [22, 24, 39].

#### Greater family engagement in care delivery

Family engagement is central to pediatric telemedicine because caregivers often manage symptom observation, physiologic measurements, device use, behavioral interventions, and communication with clinicians [16, 38]. Caregivers may collect physiologic data, upload information, and interact with monitoring systems to support home-based care [16].

Digital interventions increasingly promote collaborative family participation. The SMARTFAMILY program involved families in shared lifestyle goals and progress monitoring through a common platform [25]. In T1D, cloud-based CGM and insulin-pump systems enable caregivers and clinicians to monitor glycemic trends and treatment adherence remotely [11, 17]. Real-time CGM sharing also improves caregiver involvement in diabetes management [45]. Parent-mediated telehealth interventions have additionally been applied in ASD [30], obesity, tele-nutrition, and home-care pathways [22, 39, 46].

Although family participation can strengthen shared-care models, it may also increase caregiver burden related to monitoring, data interpretation, and alert management [16, 38].

## Clinical applications in pediatrics

Telemedicine has progressively expanded across multiple pediatric settings, supporting follow-up and continuity of care beyond hospital-based models.

### Primary care and low-complexity acute conditions

Pediatric primary care is among the best-studied telemedicine settings. McConnochie et al. [47] found agreement between telehealth and face-to-face evaluations in 86% of cases, with highest discrepancies involving upper respiratory-ear pathology. Respiratory, gastrointestinal, genitourinary, behavioral, and skin conditions showed comparable diagnostic accuracy through telehealth.

Video consultations also demonstrated good reliability in evaluating respiratory symptoms and pediatric respiratory distress [48, 49]. Telemedicine integrated into schools and childcare settings improves continuity of care, reduces transportation barriers, and supports underserved populations [40].

Remote examination devices such as TytoCare have further reduced the gap between virtual and in-person care. Studies reported high concordance for heart, lung, and skin findings, although lower agreement remained for otoscopy and throat examinations [47, 50, 51]. Mobile medical devices also supported early discharge and remote monitoring after hospitalization [52], while pediatricians recognized tele-homecare as useful for improving hospital experience and reducing hospitalization length despite concerns regarding workload and suitability [53].

### Children with chronic conditions and medical complexity

Children with chronic diseases and medical complexity require continuous coordinated care and frequent monitoring [54]. Structured telemedicine programs reduced acute care utilization and improved family confidence in home management [55], while telehealth-supported coordination improved caregiver perception of care quality and family-centeredness [56].

Telemedicine is especially valuable for technologically dependent children. TytoHome proved feasible and acceptable in children on long-term mechanical ventilation, reducing outpatient visits without compromising oversight [57]. A systematic review including chronic conditions such as obesity, asthma, T1D, ADHD, cystic fibrosis, and skin disorders found telehealth outcomes comparable or superior to standard care regarding adherence, symptom control, and quality of life [58]. Similar findings were confirmed across pediatric chronic diseases [5]. In children with medical complexity, telehealth was associated with fewer hospital admissions, ED visits, and healthcare costs, together with high caregiver satisfaction [59].

### Pediatric subspecialties

#### Endocrinology and diabetology

Pediatric diabetology has one of the more mature evidence bases for telehealth, particularly when follow-up is integrated with CGM and insulin-pump systems. School-centered telemedicine programs improved HbA1c and reduced emergency healthcare use in T1D [60]. Telemedicine follow-up was non-inferior to face-to-face care while reducing patient burden [61], and remote glucose monitoring in newly diagnosed children improved glycemic outcomes [62]. A recent review confirmed benefits on glycemic control, ketoacidosis reduction, and patient satisfaction, especially when integrated with CGM and insulin-pump systems [63]. Nevertheless, evidence remains context-dependent, and sustained benefit depends on device access, data-sharing workflows, family capacity, and in-person assessment when clinically required.

Telemedicine also improved attendance for growth, thyroid, and reproductive endocrinology consultations in rural populations [64, 65].

#### Obesity

Pediatric obesity management is well suited to telehealth because it requires frequent follow-up, multidisciplinary support, and family engagement. Telemedicine interventions improved BMI-related outcomes compared with standard care [66], while long-term telehealth lifestyle programs achieved BMI reductions comparable to in-person interventions [67]. Hybrid interventions combining face-to-face and remote support improved BMI and family involvement [68, 69]. Digital tools may enhance engagement; platforms integrating mHealth, artificial intelligence, serious games, and lifestyle applications improved adherence and participation more effectively than passive teleconsultation alone [70, 71]. However, heterogeneity in interventions, outcomes, and follow-up duration limits definitive comparisons across programs.

#### Pulmonology

Asthma is one of the most widely investigated areas of pulmonary telehealth in pediatrics [72]. School-based telehealth programs, often supported by trained nurses or healthcare providers, have been associated with improved symptom control, more symptom-free days, and reduced healthcare utilization in children with moderate-to-severe asthma [73]. These benefits appear particularly relevant in underserved urban populations, where telemedicine can support medication management, reinforce treatment adherence, and reduce barriers to regular follow-up care [74–76]. Evidence is relatively mature for structured chronic-care and school-based pathways, whereas wearable-enabled monitoring and AI-supported approaches remain emerging.

In cystic fibrosis, telemedicine has also shown promising results. Mobile health applications, remote monitoring platforms, and home spirometry have been used to improve treatment adherence, support clinical follow-up, and enhance patients’ quality of life [77]. Studies suggest that telemedicine combined with home spirometry can achieve outcomes comparable to traditional in-person visits, while maintaining high satisfaction among patients and caregivers [78].

#### Cardiology

Telemedicine in pediatric cardiology is mainly useful for follow-up and rhythm monitoring, although some conditions still require direct examination and imaging [79]. Telehealth proved safe and feasible for arrhythmias, cardiomyopathies, and postoperative congenital heart disease follow-up [81]. Telecardiology networks enabled ECG transmission and coordinated multidisciplinary care between hospitals and community providers [80–82].

Fetal tele-echocardiography allows locally acquired images to be interpreted remotely by pediatric cardiologists, supporting prenatal identification of complex congenital heart disease, parental counseling, and delivery planning [83, 84].

Remote monitoring programs for high-risk congenital heart disease reduced complications and improved growth outcomes [85]. Interstage home monitoring for infants with single-ventricle physiology may combine oxygen saturation, weight, feeding, symptoms, and video consultations to facilitate early recognition of clinical deterioration and timely escalation of care [85, 86].

Telehealth may also support selected components of pulmonary hypertension follow-up, including symptom assessment, medication management, oxygen saturation monitoring, and review of diagnostic results. However, evidence in pediatric populations remains limited, and periodic in-person clinical, echocardiographic, laboratory, and hemodynamic assessment remains necessary [87].

Wearable technologies and AI are also emerging: Apple Watch ECG recordings showed excellent agreement with standard ECGs in infants [88], while AI-based ECG interpretation demonstrated high specificity for rhythm analysis [89]. Deep-learning and remote sensing technologies may further improve neonatal rhythm monitoring [90]. Remote monitoring of pacemakers and implantable cardioverter-defibrillators enables surveillance of battery status, lead integrity, arrhythmias, programmed parameters, and delivered therapies, potentially reducing selected hospital visits [91]. However, disparities persist, with lower telehealth utilization among uninsured and non-English-speaking families and those lacking reliable internet access [92].

#### Neurology, mental health, and child neuropsychiatry

Tele-neurology has demonstrated broad feasibility in the management of conditions such as epilepsy, headache disorders, ADHD, autism spectrum disorder (ASD), neuromuscular diseases, and movement disorders. Large-scale studies have shown that telemedicine is clinically appropriate in approximately 95% of cases, with very few adverse events reported [93, 94]. Nevertheless, appropriateness varies by condition and visit purpose, and in-person examination remains necessary when neurological assessment, safeguarding, diagnostic testing, or acute deterioration cannot be addressed remotely.

Telemedicine has also improved access to care for underserved and rural populations, reducing hospitalizations, travel burden, and caregiver strain [95, 96]. In headache management, teleconsultations have decreased family burden and avoided unnecessary transfers to specialized centers [97, 98]. Similar benefits were observed in lower-resource settings through hub-and-spoke tele-neurology models [99], and school-based telehealth also supported ADHD follow-up [100].

Telepsychiatry has shown diagnostic reliability and clinical outcomes comparable to in-person visits [101–104], improving ADHD symptoms [105], externalizing behaviors through parent training [106], and broader mental-health outcomes in children and adolescents [107].

In ASD, parent-mediated telehealth interventions improved caregiver competencies and behavioral outcomes [108, 109], while internet-delivered cognitive-behavioral therapy (CBT) showed efficacy comparable to face-to-face therapy for anxiety, depression, obsessive–compulsive disorder, chronic pain, and sleep disorders [110–112]. Telemedicine additionally supported continuity of care in epilepsy and neurological conditions [113, 114].

In adolescents, digital interventions improved engagement, symptom control, and continuity in mental health [112, 115], while telemedicine-based family therapy and CBT showed promising outcomes in eating disorders [116, 117]. Digital programs were also associated with reduced risky behaviors, including substance use [118].

#### Neonatal, rehabilitative, and complex care

In neonatal and complex care, telemedicine may improve access to specialist expertise, continuity of care, and transition from hospital to home. Teleconsultation models reduced unnecessary transfers and improved neonatal triage [119], while hybrid tele-echocardiography supported diagnosis of congenital heart disease [120]. Remote participation in neonatal intensive care rounds also improved multidisciplinary management [121]. These applications remain comparatively less mature than diabetes or asthma telehealth and require robust safety protocols, local clinical capacity, and timely access to in-person assessment.

Remote follow-up reduced readmissions and emergency visits while maintaining high caregiver satisfaction [122]. Tele-neurology and tele-neuromonitoring enhanced neurological assessment and seizure monitoring in high-risk newborns [123–126]. Telemedicine additionally strengthened family-centered care and parental support in NICU settings [127, 128].

Remote rehabilitation accelerated therapy initiation and proved feasible in children with developmental vulnerability and disabilities [129–131].

Telemedicine may also reduce healthcare costs and optimize neonatal intensive-care resources [119, 132].

Table 1 provides a condensed narrative evidence map highlighting the most representative studies, clinical applications, study designs, and key limitations across the main pediatric telemedicine areas.

**Table 1:** Condensed narrative evidence map of pediatric telemedicine applications across clinical settings. The table presents selected representative studies, including their clinical applications, study designs, key findings, and principal limitations. It is intended to support navigation of the evidence discussed in the review and does not constitute a systematic evidence synthesis or a formal comparison or ranking of interventions. Evidence maturity and study-specific limitations are further interpreted in the accompanying narrative text

Area	Study	Population	Intervention	Key findings	Study design evidence maturity and key limitation
Primary care and acute conditions	McConnochie et al. [47]	Children with acute illness	Telemedicine vs in-person evaluation	86% agreement between telehealth and face-to-face evaluations; main discrepancies in upper respiratory-ear pathology	Comparative diagnostic study; single-setting assessment; limited generalizability to complex acute presentations
	Wagner et al. [51]	Children	Remote physical examination with mobile device	Good agreement for remote pediatric examinations	Nonrandomized controlled study; potential selection bias and short-term assessment
	Bernuzzi et al. [53]	Pediatricians	Tele-homecare implementation	Tele-homecare perceived as useful despite workload concerns	Qualitative study; perceptions do not establish clinical effectiveness
Chronic conditions and medical complexity	Notario et al. [55]	Children with medical complexity	Home-based telemedicine	Reduced acute care utilization and improved family confidence	Home-based telemedicine study; single-program design; generalizability limited
	Looman et al. [56]	Caregivers of children with medical complexity	Telehealth care coordination	Improved caregiver perception of care quality and family-centeredness	Randomized care-coordination trial; caregiver-reported outcomes and setting-specific model
	Ferro et al. [59]	Children with medical complexity	Telemedicine	Reduced hospital admissions, ED visits, and healthcare costs	Integrative review; mostly observational evidence and heterogeneous interventions
Endocrinology and diabetology	Plachy et al. [61]	Children with type 1 diabetes	Telemedicine follow-up	Non-inferior glucose control compared with face-to-face care	Randomized study; non-inferiority context and clinician time burden
	Crossen et al. [62]	Newly diagnosed children with type 1 diabetes	Remote glucose monitoring	Improved glycemic outcomes	Single-center pilot study; small sample and short follow-up
	Fogliazza et al. [63]	Children and adolescents with T1D	Telemedicine integrated with CGM and pumps	Improved glycemic control and reduced ketoacidosis	Narrative/review evidence; heterogeneous studies and limited long-term data
Obesity	Uengarporn et al. [66]	Children with obesity	Telemedicine-based obesity intervention	Improved BMI-related outcomes	Randomized controlled trial; single intervention/context and limited follow-up
	Struckmeyer et al. [67]	Children and adolescents with obesity	Structured telehealth obesity program	BMI reductions comparable to in-person care	Retrospective matched-control study; residual confounding and nonrandomized design
	Calcaterra et al. [68]	Children and adolescents with obesity	In-person vs virtual supervised combined training	Virtual supervised training emerged as a promising strategy to improve cardiometabolic health and adherence to exercise programs	Comparative supervised-training study; limited external generalizability
Pulmonology	Halterman et al. [73]	Children with moderate-to-severe asthma	School-based telehealth asthma management	Improved symptom-free days and reduced healthcare utilization	Randomized clinical trial; school-based US model may not generalize broadly
	Le et al. [74]	Children with asthma and their caregivers	Telemedicine-based educational program	Telemedicine education for caregivers improved asthma control in children compared with standard care	Randomized controlled trial; single setting and limited long-term follow-up
	Medbo et al. [78]	Patients with cystic fibrosis	Telemedicine with home spirometry	Comparable outcomes to in-person care and high satisfaction	Prospective multicenter study; no randomized comparator and selected families
Cardiology	Mannarino et al. [82]	Children with cardiac disease	Pediatric telecardiology system	Improved integration between hospitals and primary care	Implementation study; local infrastructure may limit transferability
	Harahsheh et al. [85]	Children with single-ventricle physiology	Telemedicine home monitoring	Reduced complications and improved growth outcomes	Observational home-monitoring study; historical/selection bias and single condition
	Paech et al. [88]	Pre-term neonates	Apple Watch ECG recordings	Excellent agreement with standard ECG	Accuracy study; technical validity does not establish clinical outcome benefit
	Teich et al. [89]	Pediatric patients	AI-based ECG analysis	High specificity for rhythm analysis	Algorithm development study; requires external validation and regulatory assessment
	Brown and Holland [84]	Fetuses at risk of congenital heart disease	Fetal tele-echocardiography	Supported remote prenatal diagnosis, counseling, and delivery planning	Single-center implementation study; dependent on trained local image acquisition and specialist interpretation
	Zartner et al. [91]	Young patients with congenital heart disease and implanted devices	Remote monitoring of pacemakers and implantable cardioverter-defibrillators	Enabled surveillance of device function, lead integrity, arrhythmias, and delivered therapies	Observational device-monitoring study; selected population and older technological context
Neurology and neuropsychiatry	Kaufman et al. [94]	Pediatric neurology patients	Child neurology telemedicine	Clinically appropriate in most encounters	Large observational encounter analysis; pandemic-period selection and no comparator
	Fischer-Grote et al. [103]	Children and adolescents	Online and remote mental-health interventions	Effective after COVID-19 pandemic	Systematic review/meta-analysis; heterogeneous interventions and outcomes
	Myers et al. [105]	Children with ADHD	Telehealth behavioral and pharmacological care	Reduced ADHD symptom severity	Community-based randomized trial; model-specific implementation and follow-up limits
	Vismara et al. [108]	Children with autism	Telehealth parent training	Improved caregiver competencies and behavioral outcomes	Randomized parent-training study; modest sample and intervention-specific findings
	Lenhard et al. 2017 [110]	Adolescents with OCD	Internet-delivered CBT	Significant clinical improvement	Randomized controlled trial; condition-specific digital CBT and selected participants
Neonatal, rehabilitative, and complex care	Jagarapu et al. [119]	Neonates in NICU	TeleNICU	Reduced unnecessary transfers	Review/implementation evidence; neonatal triage models remain heterogeneous
	Sarik et al. [122]	NICU infants and caregivers	Telehealth follow-up	Reduced readmissions and emergency visits	Follow-up program study; selection bias and limited comparative safety data
	Variane et al. [123]	Neonates	Integrated tele-neurocritical care	Proposed integrated tele-neurology model	Tele-neurology/monitoring study; emerging application with limited prospective outcomes
	Pineda et al. [130]	Preterm infants	Telehealth early intervention	Reduced time to therapy initiation	Rehabilitation study; feasibility-focused and variable developmental populations

The table includes selected representative studies for each clinical area. Additional studies and references are discussed in the main text. Evidence-maturity descriptions are narrative and do not constitute formal evidence grading

*ADHD* attention-deficit/hyperactivity disorder, *ASD* autism spectrum disorder, *CBT* cognitive behavioral therapy, *CGM* continuous glucose monitoring, *ED* emergency department, *NICU* neonatal intensive care

## Benefits and opportunities

The potential benefits of pediatric telemedicine differ by patient group and care pathway. In rural or underserved populations, hub-and-spoke models may improve access to specialist input; for children with medical complexity, structured home-based follow-up may reduce travel and support coordination; in chronic disease, remote data review can reinforce self-management; and in school-based or post-discharge pathways, telehealth may reduce logistical barriers [10, 11, 13, 16, 26, 39].

Telehealth can facilitate access to pediatric specialists through remote consultations and hub-and-spoke models, allowing tertiary centers and multidisciplinary teams to support local services [30, 38]. It may also reduce transportation costs, missed workdays, school absenteeism, and logistical stress; in telehomecare programs, many families preferred home hospitalization over conventional admission [12]. These advantages should not be interpreted as evidence of superior clinical outcomes in every setting.

Remote-monitoring systems may support earlier recognition of concerning trends, personalized follow-up, and treatment satisfaction when data are clinically validated, reviewed within a defined workflow, and linked to action plans [11, 16, 45]. They may also enhance flexibility and autonomy in daily routines [43], but false alerts, caregiver burden, clinician workload, and unequal access must be considered.

Telemedicine may optimize healthcare resources by reducing selected visits, interfacility transfers, and hospital use [12, 34, 38, 133]. Economic effects are sensitive to program design, reimbursement, technology costs, staff time, and local service configuration; the available evidence does not support universal cost-effectiveness claims [44]. Overall, telemedicine is best viewed as a complementary service model whose value should be assessed within specific pediatric pathways.

## Limits and challenges

Pediatric telemedicine presents important clinical, technological, organizational, and ethical challenges. A complete physical examination, including auscultation, palpation, neurological assessment, growth monitoring, and developmental evaluation, cannot always be performed reliably at a distance. Telemedicine should not substitute for in-person assessment when direct examination is required for safe diagnosis or management, particularly in young or uncooperative children and in potentially acute conditions [14, 38].

Digital inequity remains a major concern. Effective telemedicine depends on stable internet access, adequate devices, private space, affordable data, language support, and sufficient digital literacy. Families with socioeconomic difficulties or limited access to technology may therefore experience reduced access to telehealth services, potentially worsening healthcare disparities [33, 92, 134]. These barriers may be particularly pronounced in low- and middle-income settings, where asynchronous telemedicine, smartphone-based platforms, low-bandwidth communication systems, task-sharing with trained local healthcare workers, and context-adapted hub-and-spoke referral networks may improve feasibility and access.

The central role of caregivers can represent both an advantage and a burden. Parents are often responsible for symptom reporting, remote examinations, device management, and communication with clinicians, increasing responsibility and stress, particularly in chronic and medically complex conditions [16, 38]. Programs should monitor caregiver burden and provide training, technical support, and clear routes for escalation.

Organizational barriers further limit implementation. Incomplete integration with electronic health records may cause fragmented workflows, duplicated documentation, and increased clinician workload [11, 16]. Sustainable implementation requires clear eligibility and triage protocols, staff training, documentation standards, reimbursement models, technical support, escalation pathways, and explicit responsibility for reviewing data and arranging follow-up.

Privacy, cybersecurity, safeguarding, and medico-legal concerns are particularly relevant in pediatrics because telemedicine involves transmission of sensitive health information and patient-generated data. Services should address identity verification, consent or assent where appropriate, adolescent confidentiality and privacy at home, safeguarding disclosures, secure data handling, cross-border licensure, documentation of decisions, and emergency deterioration during or after a virtual visit [4, 33].

In addition, available evidence remains heterogeneous, with many studies involving small populations and short follow-up. Long-term effectiveness, safety, cost-effectiveness, equity, and validation of AI-based systems still require further research [4, 18]. Overall, telemedicine should remain clinically appropriate, equitable, and integrated with face-to-face care when necessary.

## Future perspectives

The future of pediatric telemedicine will depend on stable integration into routine care through hybrid models combining remote and in-person visits according to clinical complexity and family needs. Priority areas include validated pediatric remote-examination standards, pediatric tele-triage algorithms, and hybrid pathways that specify when in-person assessment is necessary.

Remote monitoring and patient-generated data may support more personalized management by enabling continuous assessment of symptoms, adherence, and physiological parameters. Broader implementation will require standardized protocols, interoperability standards, data governance, privacy safeguards, and equity metrics that monitor access and outcomes across socioeconomic, language, geographic, and disability-related differences.

Training is essential. Healthcare professionals need skills in remote assessment, digital communication, triage, documentation, safeguarding, and digital governance, while families require guidance to use telemedicine tools safely, recognize red flags, and access urgent care. Further research should include prospective safety studies, caregiver-burden measurement, cost-effectiveness analyses, and independent AI validation in diverse pediatric populations.

**Fig. 1:**
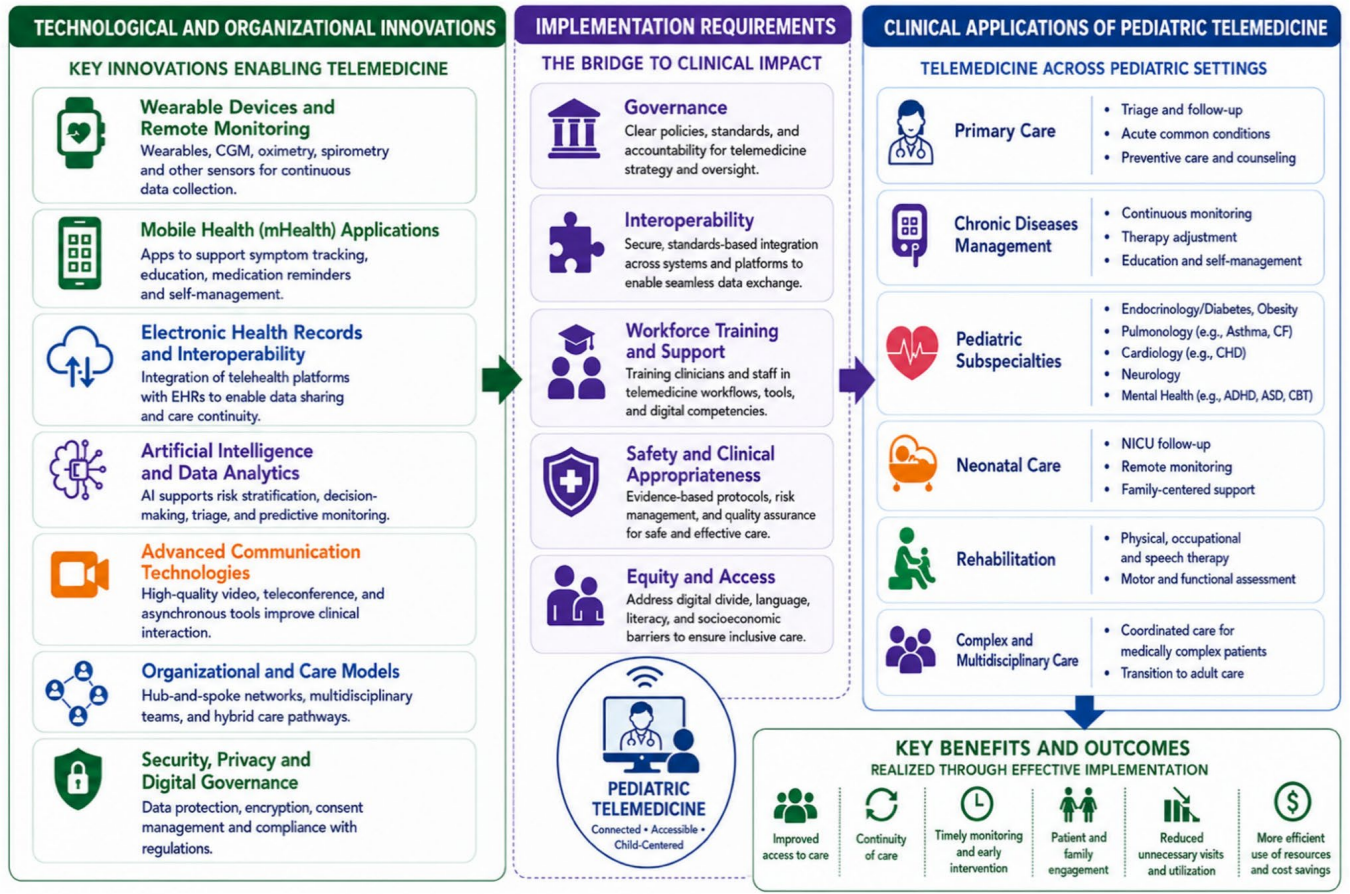
Conceptual overview of pediatric telemedicine, showing technological and organizational innovations, implementation requirements, clinical applications, and key benefits and outcomes. The figure was generated with ChatGPT 5.5 as a visual drafting aid, then critically reviewed, revised, and verified by all authors. It is conceptual and does not present quantitative or unsupported claims. *ADHD* attention-deficit/hyperactivity disorder, *AI* artificial intelligence, *ASD* autism spectrum disorder, *CBT* cognitive behavioral therapy, *CF* cystic fibrosis, *CGM* continuous glucose monitoring, *CHD* congenital heart disease, *EHR* electronic health record, *mHealth* mobile health, *NICU* neonatal intensive care unit

**Fig. 2:**
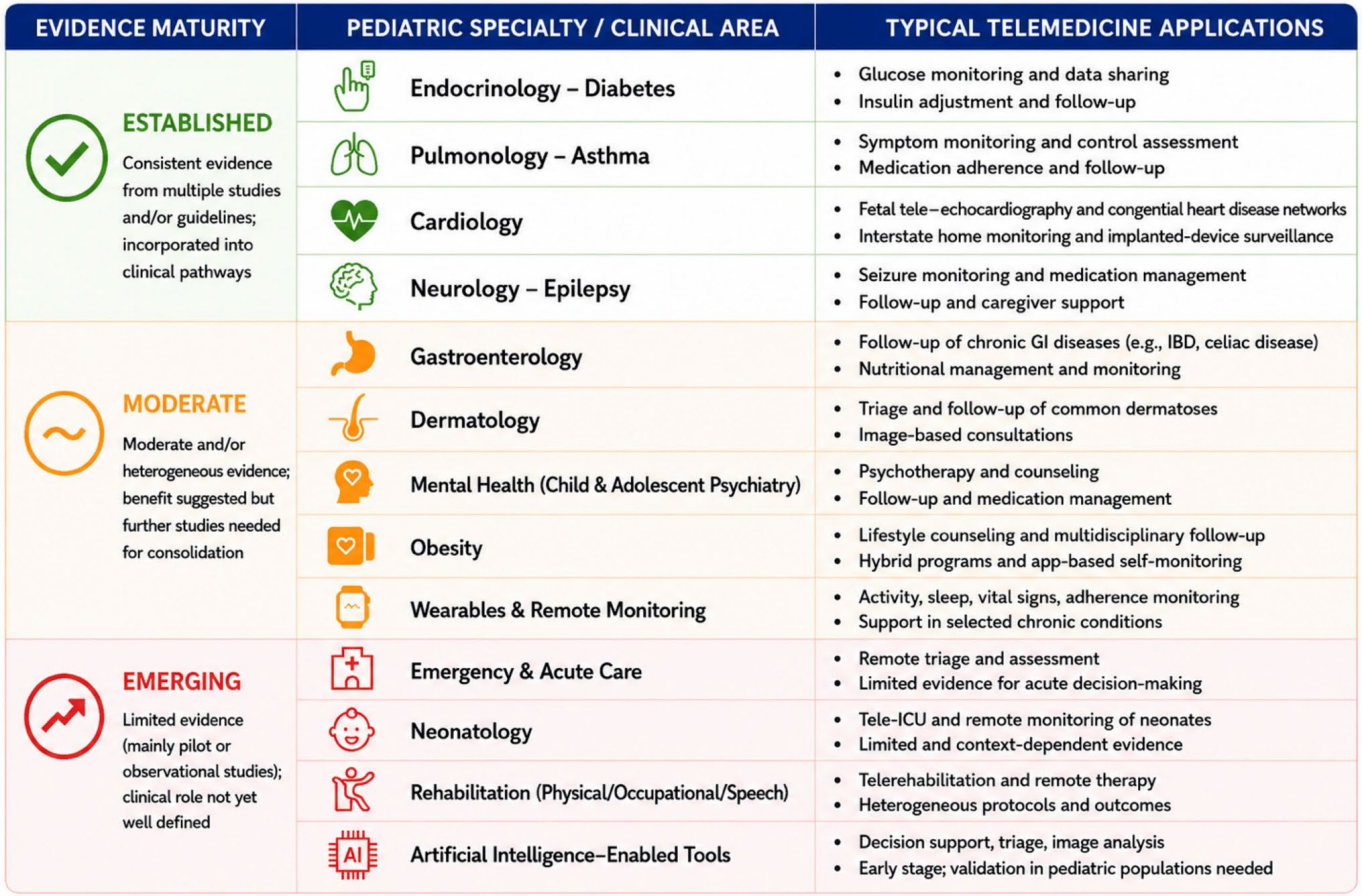
Visual summary of evidence maturity across the main pediatric telemedicine specialties. The figure was generated with ChatGPT 5.5 as a visual drafting aid, then critically reviewed, revised, and verified by all authors. *AI* artificial intelligence, *GI* gastrointestinal, *IBD* inflammatory bowel disease, *ICU* intensive care unit

Figure 1 provides a conceptual overview of technological and organizational innovations, implementation requirements, clinical applications, and the key benefits and outcomes of pediatric telemedicine. Figure 2 complements this overview by summarizing the maturity of the available evidence across the main pediatric specialties as established, moderate, or emerging.

## Conclusions

Telemedicine is increasingly integrated into pediatric care as a complementary option for selected patients and clinical pathways, particularly for follow-up and chronic-care models, rather than as a substitute for in-person assessment. However, the heterogeneous literature reviewed here does not support assuming improved clinical outcomes across all pediatric settings.

Technological and organizational innovations, including hybrid care and digital follow-up systems, may expand opportunities for personalized pediatric care. Their implementation remains limited by incomplete remote examination, digital inequities, caregiver burden, fragmented systems, privacy concerns, and variable evidence quality. As a narrative review, this article provides a cross-specialty critical overview rather than a formal quantitative estimate of effectiveness or certainty.

Future progress will require standardized protocols, interoperable infrastructures, appropriate professional and family training, and stronger evidence on long-term safety, effectiveness, cost-effectiveness, equity, and sustainability. Ultimately, the future success of pediatric telemedicine will depend not only on technological advances, but also on its careful integration into clinically appropriate, equitable, interoperable, and family-centered models of care.

## Data Availability

No datasets were generated or analysed during the current study.

## Abbreviations

ADHD: Attention-deficit/hyperactivity disorder
AI: Artificial intelligence
ASD: Autism spectrum disorder
BMI: Body mass index
CBT: Cognitive behavioral therapy
CF: Cystic fibrosis
CGM: Continuous glucose monitoring
CHD: Congenital heart disease
DHI: Digital health interventions
ECG: Electrocardiogram
ED: Emergency department
EHR: Electronic health record
HIE: Hypoxic–ischemic encephalopathy
ICT: Information and communication technologies
mHealth: Mobile health
NICU: Neonatal intensive care unit
PGHD: Patient-generated health data
QoL: Quality of life
RCT: Randomized controlled trial
RPM: Remote patient monitoring
T1D: Type 1 diabetes
SaMD: Software as a medical device
